# Testing the Limits: Functional Strengths and Weaknesses of Poacher (Agonidae) Armor

**DOI:** 10.1093/iob/obag003

**Published:** 2026-01-28

**Authors:** L E Martinez, M L Vandenberg, K E Cohen, A P Summers, C M Donatelli

**Affiliations:** Department of Biology, Rhodes College, Memphis, TN 38112, USA; Friday Harbor Laboratories, University of Washington, Friday Harbor, WA 98250, USA; Department of Biology, University of Washington, Seattle, WA 98195, USA; Friday Harbor Laboratories, University of Washington, Friday Harbor, WA 98250, USA; Department of Biology, California State University, Fullerton, CA 92831, USA; Friday Harbor Laboratories, University of Washington, Friday Harbor, WA 98250, USA; Department of Biology, University of Washington, Seattle, WA 98195, USA; School of Engineering and Technology, University of Washington, Tacoma, WA 98402, USA

## Abstract

Dermal armor serves a variety of functions across animal lineages including defense, offense, display, and prehension. Small differences in armor structure, plate size, or overlap may complement large differences in behavior or ecology. We characterized damage to an armored fish—the gray starsnout poacher (*Bathyagonus alascanus*) to probe whether there are differences in plate function within a single species. We quantified damage to poacher armor and skeleton under different force modes, including crushing, puncture, abrasion, and blunt impact, using micro-computed tomography, scanning electron microscopy, and material testing. Armor in the posterior region of the fish can withstand higher stress during crushing, suggesting they are well protected while fleeing from a crushing predator. It takes more work to puncture the anterior armor, perhaps poachers tend to face an animal threatening a puncturing attack. The dorsal plate spines are often eroded away from abrasion and/or blunt impact; we posit that the spineless ventral plates are smooth because strong sub-tidal currents cause collisions with a rocky substrate that would quickly destroy ventral spines if the plates were so equipped. The imbricated armor of *B. alascanus* has a diversity of performance against different threats, and this varies with location.

## Introduction

Dermal armor has convergently evolved in vertebrates and serves a variety of functions including locomotion, camouflage, hydrodynamics, and defense (Bruet et al. 2008; Porter et al. 2013; Kruppert et al. 2020). Our interest is in the defensive functions of heavy plates of bone that cover the body of fishes (Yang et al. 2013). The assaults on armor can take many forms including puncture, impact, crushing, and abrasion. These force modes represent distinct ecological threats that shape defensive armors to combat their consequential damages. For example, in poachers (Agonidae), the head is more heavily armored because these fish engage in head-to-head combat over territory (Kruppert et al. 2020; Vandenberg et al. 2024). Furthermore, the force mode posed by prevalent pressures can be assessed by examining the armor closely to see the marks left behind. For example, the tips of the conical armor in the pacific spiny lumpsucker (*Eumicrotremus orbis*) bear the scars of bouncing around the intertidal, and the benthic northern spearnose poacher (*Agonopsis vulsa*) shows extensive abrasion damage on the belly, and chipping of plates in areas where combat would lead to impact (Kruppert et al. 2020; Woodruff et al. 2022).

In a world of sharp-toothed predators, armor specialized to resist puncture must deflect the sharp tip or distribute the force so the local strains do not exceed the strength of the material (Bruet et al. 2008; Vernerey and Barthelat 2010; Martini and Barthelat 2016; Speck and Speck 2019). Smooth plates can cause a sharp point to slide, while a rough surface will grab that point and ensure the puncture force is distributed over the area of the entire plate (Liu et al. 2018).

The material of the armor also plays a role in dissipating the energy of puncture or impact, with progressive failure and crack containment serving to increase the energy required to do deep damage (Song et al. 2011; Zhu et al. 2012). For example, the material in the smooth armor plates of the gray bichir (*Polypterus senegalensis*) is four layers of ganoine, dentine, isopedine, and bone which redistributes stress and prevents radial cracking by dissipating energy through thick layers that differ in material properties (Bruet et al. 2008). Materials inspired by the shape and structure of biological armors, such as fracture resistant ceramics and flexible lightweight body armor, are capable of resisting ballistic projectile penetration (Martini and Barthelat 2016; Speck and Speck 2019; Yaseen et al. 2021).

Armored fishes also encounter crushing forces from both biotic and abiotic factors, such as blunt teeth and crab claws as well as pressure from squeezing beneath rocks and into crevices (Lemopoulos and Montoya-Burgos 2021; Muruga et al. 2022). Delocalized compressive forces such as crushing can result in catastrophic local bending of individual armor plates, or equally catastrophic buckling of the armor. These two failure modes, bending and buckling, are resisted with different design parameters. Bending is minimized by keeping armor plate lengths very small, which leads to many plates with associated joints (Vernerey and Barthelat 2010). Resistance to buckling decreases as the number of joints increase, causing structures to topple rather than maintain rigidity, thus the plate size cannot protect against both modes of failure (Euler Column Buckling). For example, the tail of a seahorse has just four plates, making it difficult to buckle. In contrast, the bichir has more than 26 around the circumference at the dorsal fin, and each individual scale resists bending, while the overlapping arrangement allows large deformations of the body cross section, which power ventilation (Graham et al. 2014). Engineered ceramic and silicone composites inspired by fish armor similarly use highly imbricated short plates that provide compressive and bending resistance while still allowing for flexibility due to the high degree of overlap (Yang et al. 2013; Martini and Barthelat 2016).

Many armored fishes are benthic or reef associated, constantly interacting with abrasive surfaces such as gravel, rocks, and corals (Lemopoulos and Montoya-Burgos 2021). Collisions due to strong subtidal currents can cause impact forces that may spall or chip structural complexities on the armor surface (Woodruff et al. 2022). Such damages caused by blunt impact forces generally scale inversely with the plasticity of the damaged tissue (Ressel et al. 2016). Additionally, frequent collisions may gradually erode armor leading to degradation and increased vulnerability (Amini and Miserez 2013). Resistance to abrasion requires a hard material with few local protrusions so that contact with the abrasive surface is spread out over a larger area (Amini and Miserez 2013). An example of this is the differing morphology of the dorsal and ventral plates in the armor of the Northern spearnose poacher (*Agonopsis vulsa*). Their dorsal armor plates feature larger spines, while their ventral plates that are in constant contact with the substrate are flat and distribute abrasive assault over a greater area (Kruppert et al. 2020).

**Fig. 1:**
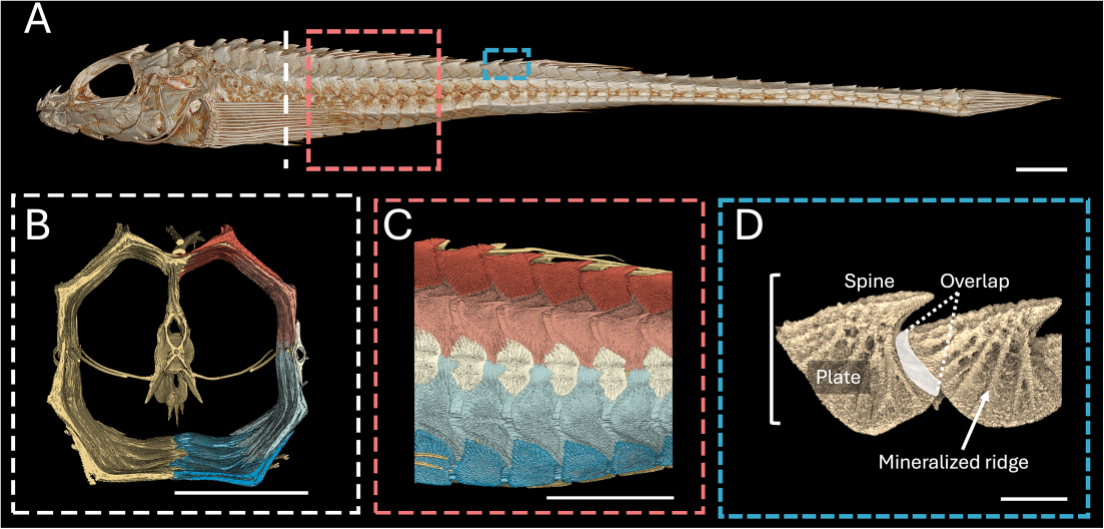
Armor structure and external plate morphology of dissected poacher plates, scale bars measure 5 mm. Plates are segmented and colored along rows with dorsal plates in red, lateral plates in pink and light blue, rail plates in white, and ventral plates in dark blue. (A) Lateral μCT visualization of *Bathyagonus alascanus*. (B) Cross-sectional μCT visualization of segmented armor rows. (C) Lateral μCT visualization of segmented armor rows. (D) μCT lateral view of two plates identifying key anatomical structures.

Poachers are a family of heavily armored benthic fish with 54 species and 24 genera with the majority in the north Pacific, and some in the southern temperate Pacific and north Atlantic (Kanayama 1991; Smith and Busby 2014). Their bony plates are arranged in eight rows that provide coverage in an octagonal shape around the body (Fig. 1A–D). In most species, this octagonal cross section transitions to six rows posterior to the dorsal fin (Busby 1998). Unlike most fishes, poachers invest heavily in their armor, on average having five times more mineralization in the plates than in their skeleton (Kruppert et al. 2020). Heavy armor comes at a metabolic cost making locomotion more inefficient (Kruppert et al. 2020). Adults have denser armor, likely for benthic protection, while juveniles are pelagic and have less dense armor, indicating a change in armor function across ontogeny (Kolmann et al. 2020). Although we know that poacher armor is naturally damaged throughout their lives, little is known about the ecological challenges that such robust armor combats. Understanding how armor functions under different force modes (i.e., puncture, crushing, abrasion, and impact) can give a window into utility and thus, by inference, to selective pressures. The gray starsnout poacher (*Bathyagonus alascanus*) is commonly found in the Salish Sea, readily trawled in 40–100 m of water, typically dwelling on mud and shell hash (Eschmeyer and Herald 1983). *Bathyagonus alascanus* are moderate in size, armor investment, and crypsis compared to other poachers, and are abundant in the Salish Sea with little known about their ecology, making them a good model to investigate potential armor function (Kruppert et al. 2020).

Here, we investigated the damage response of *B. alascanus* armor to better understand the relationship between its structure and function. We aim to catalog damage from biotic and abiotic factors and investigate the strengths and weaknesses of poacher armor in adults. The goals of our study are three-fold: (1) quantify the damage to poacher armor from crushing, puncture, abrasion, and impact using a material testing system and a rotary grinder; (2) quantify the strengths of the armor plates by comparing the force applied and breakage observed and by comparing the degrees of erosion on the armor; and (3) compare strengths and weaknesses across the body to test for regions with specialized functionality.

## Methods

### Specimen collection and preparation

We collected 19 gray starsnout poachers (74–132 mm in total length) in trawls on the Friday Harbor Laboratories’ RV Kittiwake in the San Juan Channel. Captured fish were housed in flow-through tanks at Friday Harbor Laboratories prior to euthanasia with methanesulfonate (MS-222) following IACUC protocol #4238–03 before freezing specimens in individual bags until they were thawed for testing. All specimens were limited to one freeze-thaw cycle. Using frozen specimens has been shown to have little effect on bone material properties and furthermore, we did not compare fresh to frozen specimens (Kaye 2012). We used five specimens for each damage test excluding impact which only had four specimens due to fish availability.

### Puncture (teeth) and crushing (crab claw)

**Fig. 2:**
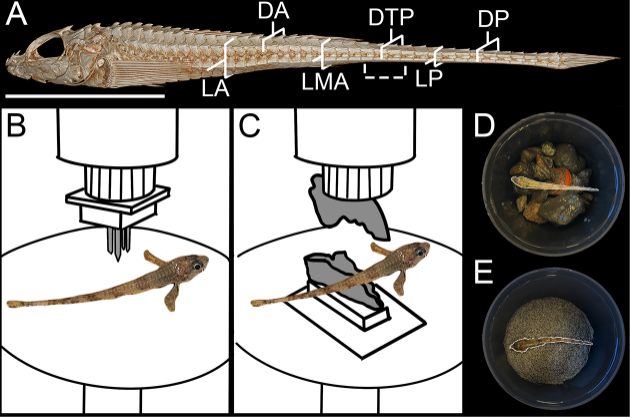
Damage testing methods. (A) μCT scan of *B. alascanus* showing the locations along the body used for MTS trials (solid brackets), and the tested region for blunt impact and abrasion quantification (dashed bracket). The scale bar measures 25 mm. MTS line drawings showing (B) a puncture trial performed dorsally between the two dorsal fins and (C) a crushing trial performed dorsally between the two dorsal fins. (D) A poacher in the tumbler with rocks for impact testing. (E) A poacher in the tumbler with sand for abrasion testing. The poachers inside tumblers (C, D) have been highlighted for visibility.

To replicate puncture damage, we modeled a lingcod (*Ophiodon elongatus*) bite. We modeled the tooth arrangement from a micro-computed tomography (μCT) scan (courtesy of Kellen Hardcastle) and used nails in place of teeth. Nails serve as a good model for lingcod oral teeth, as they are kept extremely sharp through regular replacement (Carr et al. 2021). We printed a cylindrical attachment capped by a square platform measuring 3.4 cm^2^ to serve as a base for the puncture tool. We inserted the puncture tool to the movable cross head of a Materials Testing System (MTS; Synergie 100 MTS Systems Corp., Eden Prairie, MN, USA) Universal Testing Machine while the specimens rested on a flat lower platform (Fig. 2B). The platform was customized to the MTS using a Prusa XL Printer (Prusa, 2023). For each experiment, we used aluminum foil to position and hold the poacher body in place. To investigate differences in damage resistance across the poacher body, we tested six different locations: laterally parallel to the 1st dorsal fin (LA), laterally parallel to the 2nd dorsal fin (LMA), on the side of the tail (LP), dorsal scales between the dorsal fins (DA), dorsally at the point of plate transition (DTP), and at the top of the tail (DP) (Fig. 2A). We chose locations to be spaced out along the body region mainly responsible for locomotion. Additionally, half of the locations were located in the tail region where there is only one dorsal row and one ventral row of plates, rather than two of each. For each location, we measured the height of the fish and set the center nail of the puncture tool to be barely touching the top of the fish before displacing two-thirds of the measured fish height at that location.

For crushing, We used 3D Slicer (Fedorov et al. 2012) and the SlicerMorph extension (Rolfe et al. 2021) to segment the upper and lower claws of a μCT scan of a red rock crab (*Cancer productus*). Each claw was exported as an STL and scaled to four-times life size. Even scaled up, the 3D printed claw remained within reasonable size for conspecific crabs (Yamada and Groth 2016). We added a cylindrical attachment to the upper claw and a platform attachment to the lower claw in Tinkercad (Autodesk, 2024). We printed each claw with an 80% infill on a Prusa XL 3D printer in PLA. The cylindrical upper claw attachment directly fit into the movable crosshead of the MTS, while we clamped the lower claw platform to the MTS (Fig. 2C). We tested at the same locations along the body using the same methodology as previously described for our puncture trials.

Both puncture and crushing trials were conducted using a 500N load cell and a displacement speed of 100 mm/min. We recorded and saved a time series of both displacement (mm) and force (N) from the start to end of the trial (Tables 1 and 2).

**Table 1:** Average extension, maximum force, and work to maximum force for all puncture trials (*n* = 30), grouped by trial location along the fish

Trial location	Extension (mm)	Max force (N)	Work to max force (mJ)
LA	5.72 ± 0.30	12.64 ± 1.77	23.63 ± 6.18
DA	4.52 ± 0.30	10.76 ± 2.56	19.08 ± 7.14
LMA	4.34 ± 0.46	13.62 ± 3.80	21.42 ± 4.73
DPT	2.75 ± 0.18	10.41 ± 1.72	9.15 ± 3.55
LP	2.93 ± 0.49	8.64 ± 2.02	11.57 ± 3.99
DP	2.06 ± 0.14	7.85 ± 3.07	5.54 ± 2.24

**Table 2:** Average extension, maximum force, stress, and work to maximum force for all crushing trials (*n* = 29), grouped by trial location along the fish

Trial location	Extension (mm)	Max force (N)	Stress (N/mm^2^)	Work to max force (mJ)
LA	6.19 ± 0.52	48.19 ± 12.09	0.72 ± 0.08	107.73 ± 41.01
DA	4.81 ± 0.84	50.01 ± 19.04	1.30 ± 0.44	80.69 ± 42.78
LMA	4.46 ± 0.35	38.37 ± 19.78	1.13 ± 0.45	59.46 ± 24.41
DPT	3.01 ± 0.27	32.76 ± 14.48	2.18 ± 0.68	32.92 ± 17.72
LP	3.21 ± 0.30	25.89 ± 7.31	1.55 ± 0.43	32.92 ± 11.66
DP	2.47 ± 0.36	22.82 ± 9.52	2.47 ± 1.22	19.69 ± 8.89

### Impact and abrasion tests

To replicate damage from abiotic sources, we used a rock tumbler with two different substrates. In order to cause blunt impact damage, we used rocks filtered through a 12.7 mm sieve (Fig. 2D), and to replicate fine abrasive damage we used sand filtered through a .507 mm sieve (Fig. 2E). We first filled one-third of the tumbler with rocks or sand, then placed the specimen inside, then filled one-third again with rocks or sand, before covering the composite with water (Fig. 2D and E). For each specimen, we tumbled the jars for 15 min before stopping it, resetting the experiment with the specimen in between the rock or sand layers, and then tumbling another 15 min. This helped ensure that specimens did not get stuck in one position in the tumbler.

### μCT scanning

Following testing for all specimens, we brought the specimens through a stepwise dehydration series from 25 to 70% ethanol solution (EtOH) in 2 h increments. Specimens were stored in 70% EtOH until scanned. We scanned the specimens using the Bruker Skyscan 1173 μCT scanner (Bruker microCT, Kontich, Belgium) at the Karel F. Liem Bio Imaging Center at Friday Harbor Laboratories in Friday Harbor, WA. Scans were captured using the parameters 65 kV, 123 μA, and 1125 ms exposure and had resolutions between 8.8 and 15.1 μm. We reconstructed the scans in NRecon (Bruker, 2005–2011) and visualized the scans in 3D Slicer ((Fedorov et al. 2012), Version 5.6.2) using the Volume Rendering and SlicerMorph ImageStacks modules (Rolfe et al. 2021).

### Scanning electron microscopy

Following μCT scanning, we dissected small segments of damaged scales for scanning electron microscopy (SEM). Scales were further dehydrated through 80–100% EtOH before being dried overnight in hexamethyldisilazane. We sputter-coated samples in gold palladium using a Cressington 108 Sputter Coater (Ted Pella, Inc.) and we imaged scales using an SEM (Neoscope JCM-5000). Using SEM, we imaged six samples: an undamaged dorsal scale, a punctured dorsal scale, a punctured lateral scale, a cracked dorsal scale, sand-eroded dorsal scales, and stone-impacted dorsal scales.

### Data analysis and statistics

To measure the angles at which fractures were formed along the plates, we used 3D Slicer and the “Markups” module to place angles along volume renders. For each fracture, we used the direction of the spine on each plate as 0°, with one leg placed along the anteroposterior axis in the plate midline, and the other leg along the fracture. Angles were measured from 0 to 180°, as angles higher than 180° would be classified on the other side of the central spine. We denoted this measurement as “Fracture angle.” To investigate differences between sand abraded and rock impacted specimens, we used 3D Slicer to measure the cross-sectional height at the plate transition point (DTP) and created segmentations of the tail twice as long as the measured height (Dashed bracket, Fig. 2A). We filled the body cavity using the Scissors and Wrap Solidify tools (Weidert et al. 2020). We used the “Segment Statistics” module to quantify segment external surface area and total volume.

We used the open source software package “R” (R Development Core Team 2008) for all data analysis. For crushed and punctured specimens, we used the CrushR repository to measure maximum force and work. Maximum force was the highest force over the load displacement curve in Newtons (N), and work was the area under the curve (mJ) (Tables 1 and 2). We corrected for size by measuring stress as the maximum force over the cross sectional area of the fish (N/mm^2^), and for punctured specimens, we measured the area under the force-displacement curve as work to maximum force (mJ).

Anterior and posterior locations were grouped based on morphological differences between the armor in the abdominal and tail regions. The anterior region has 8 rows of overlapping plates, while the posterior region has only 6. The internal vertebral morphology also differs in these regions, with abdominal vertebrae having open hemal arches and tail vertebrae having closed arches. Since these regions are morphologically distinct, we treated them as separate categorical groups rather than as continuous points along the long axis of the body.

For puncture and crush trials, each test was treated as an independent observation, as test regions were far enough apart for us to assume no mechanical interaction between regions. For these data, we performed analysis of variance (ANOVA) and bonferroni corrected pairwise *t*-tests between orientation (lateral or dorsal), location (see MTS setup description), and region (anterior or posterior) within and under the different force applications, and for abrasive and impact data we performed *t*-tests between method and surface area-to-volume ratio.

## Results

### Puncture and crushing damage

On average, poacher armor withstood the highest force during crushing trials, where the average max force was 37.2 ± 16.9N, significantly higher than 10.7 ± 3.1N in puncture tests (ANOVA, *P* < 0.001).

**Fig. 3:**
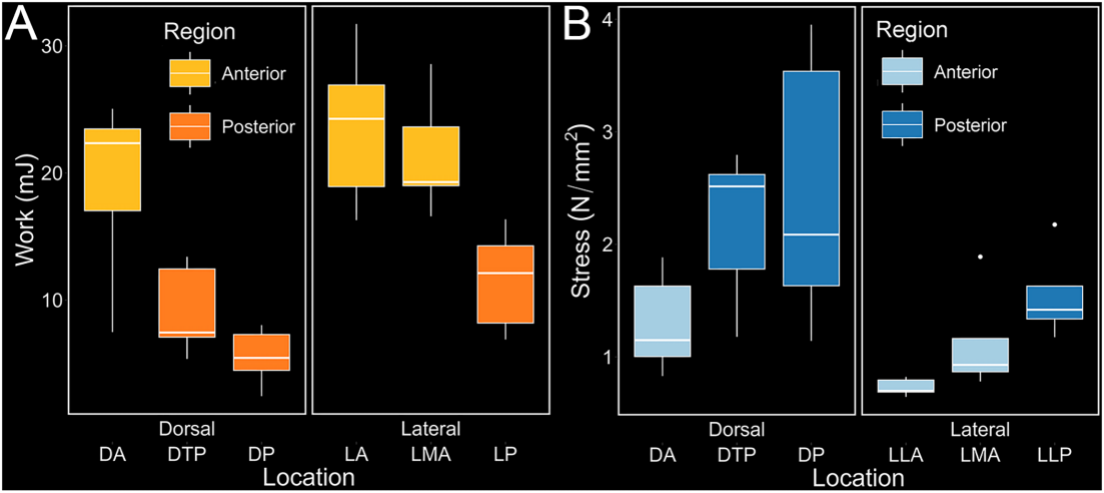
Work and stress graphs grouped by location, orientation, and region, for crushing and puncture trials. (A) Work to max force of all lateral and dorsal puncture trials, showing a significant effect of orientation on work to max force (ANOVA, *n* = 30, *P* < 0.001), location on work to max force (*P* < 0.001), and region on work to max force (*P* < 0.001). (B) Box plot stress graphs of all lateral and dorsal crushing trials, showing a significant effect of orientation on stress (ANOVA, *n* = 29, *P* < 0.001), location on stress (*P* = 0.039), and region on stress (*P* < 0.001).

For puncture trials, both orientation (ANOVA, *P* < 0.001) and location (*P* < 0.001) had significant effects on the work to maximum force (Fig. 3A). The lateral trials averaged at 18.87 ± 7.16 mJ of work compared to 11.26 ± 7.40 mJ for the dorsal trials (Table 1). Additionally, the region of the fish tested had a significant effect on work to max force (*P* < 0.001), with the anterior regions performing significantly better compared to the posterior regions, requiring on average 21.37 ± 5.96 mJ of work to reach maximum force compared to 8.75 ± 4.02 mJ (*n* = 30, pairwise *t*-test, *P* < 0.001).

For crushing trials, we examined the effects of trial location along the body and orientation of the fish (lateral or dorsal) on stress (Table 2). For dorsal tests, the average stress was higher (1.98 ± 0.94 N/mm^2^, *P* < 0.05) compared to 1.10 ± 0.47 N/mm^2^ for lateral tests, showing a significant effect of orientation on stress (Fig. 3B). When grouped by region, the opposite pattern emerged compared to puncture, with the posterior regions (DPT, LP, and DP) experiencing significantly higher stress compared to the anterior regions (LA, DA, and LMA) (2.10 ± 0.89 N/mm^2^, pairwise *t*-test *P* < 0.001, *n* = 29).

**Fig. 4:**
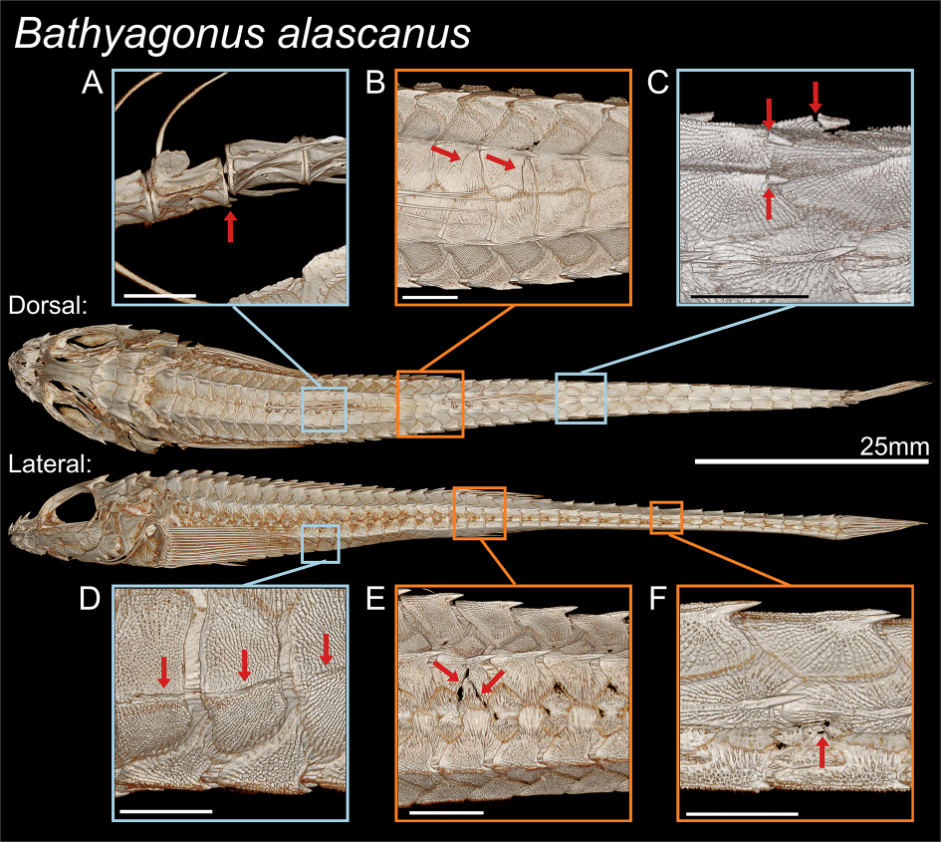
μCT scans of *B. alascanus*, previewing selected puncture and crushing damages. Arrows are used to highlight damages. Inset scale bars measure 2 mm. (A) Dislocated vertebrae due to a lateral crush parallel to the first dorsal fin. (B) Dorsal plate fractures due to a dorsal crush between the dorsal fins. (C) Broken dorsal spines due to a dorsal crush at the plate transition. (D) Cracked ventral plates due to a lateral crush parallel to the first dorsal fin. (E) Lateral plate fractures due to a lateral crush parallel to the second dorsal fin. (F) Lateral plate damage due to a lateral puncture on the side of the tail.

**Fig. 5:**
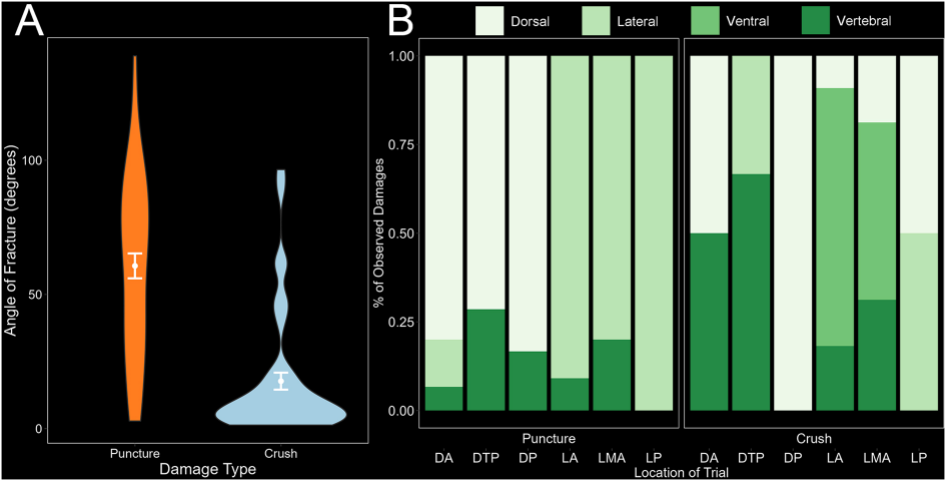
Characterization of all puncture and crushing damages. (A) Damage type had a significant effect on fracture angle (*n* = 85). Zero degrees was measured for each plate as the longitudinal direction of the spine, so that a 90° fracture angle would be perpendicular to the anteroposterior axis. (B) Observed damage locations for all puncture and crush trials (*n* = 110). Damages are classified by observed location as dorsal, lateral, ventral, or vertebral and are visualized as a proportion of all observed damages at each tested location.

Both the damage from puncture and crushing created clear fractures in the μCT scan visualizations (Fig. 4). The punctured plates all had an apparent point of puncture, and often showed fractures along the plates, coming from the point of puncture (Fig. 4E and F). Many of the crushed plates displayed a variety of damage, including fractures, cracked and dislocated vertebrae, and broken spines (Fig. 4A–D). The crushing trials yielded less variation in fracture angles, most fractures aligned closely to the 0° line along the spine (17.7 ± 23.9°), compared to the wider distribution of the puncture fracture angles (60.6 ± 33.8°), indicating that trial type had a significant effect on angle of fracture (ANOVA, *n* = 85, *P* < 0.001) (Fig. 5A). Many of the puncture fractures were primarily transverse in direction, perpendicular to the spine, while the crushed fractures were heavily concentrated along the longitudinal direction. Including vertebral damage (e.g., dislocated or chipped vertebrae), we observed 110 damages. On punctured specimens, we observed 24 lateral, 22 dorsal, 7 vertebral, and no ventral damage, while on crushed specimens we observed 4 lateral, 11 dorsal, 24 ventral, and 18 vertebral damages. For tests performed in the lateral orientation, observed damages were distributed across dorsal, ventral, lateral, and vertebral areas. For tests performed in the dorsal-ventral orientation, the majority of damage was observed on dorsal plates (Fig. 5B). From puncture, the damage appeared at the location of impact across all trial locations, while from crushing damage varied by location. LA and LMA accounted for all observed ventral damage. For dorsal trials (DA, DTP, and DP), damage was concentrated dorsally and on the vertebrae.

### Impact and abrasion damage

**Fig. 6:**
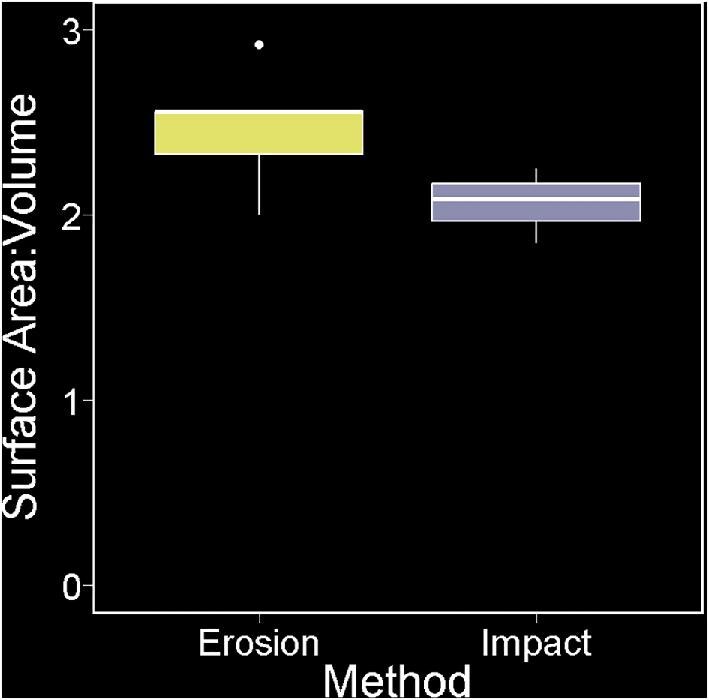
Boxplot graphs of surface area to volume ratio between sand abraded specimens (*n* = 5) and rock impacted specimens (*n* = 3).

The surface area to volume ratios of sand eroded and rock impacted specimens did not show significant difference (*t*-test, *P* = 0.075). The average surface area to volume ratio for sand eroded specimens was 2.47 mm^−^^1^ compared to 2.06 mm^−^^1^ for rock eroded specimens (Fig. 6).

**Fig. 7:**
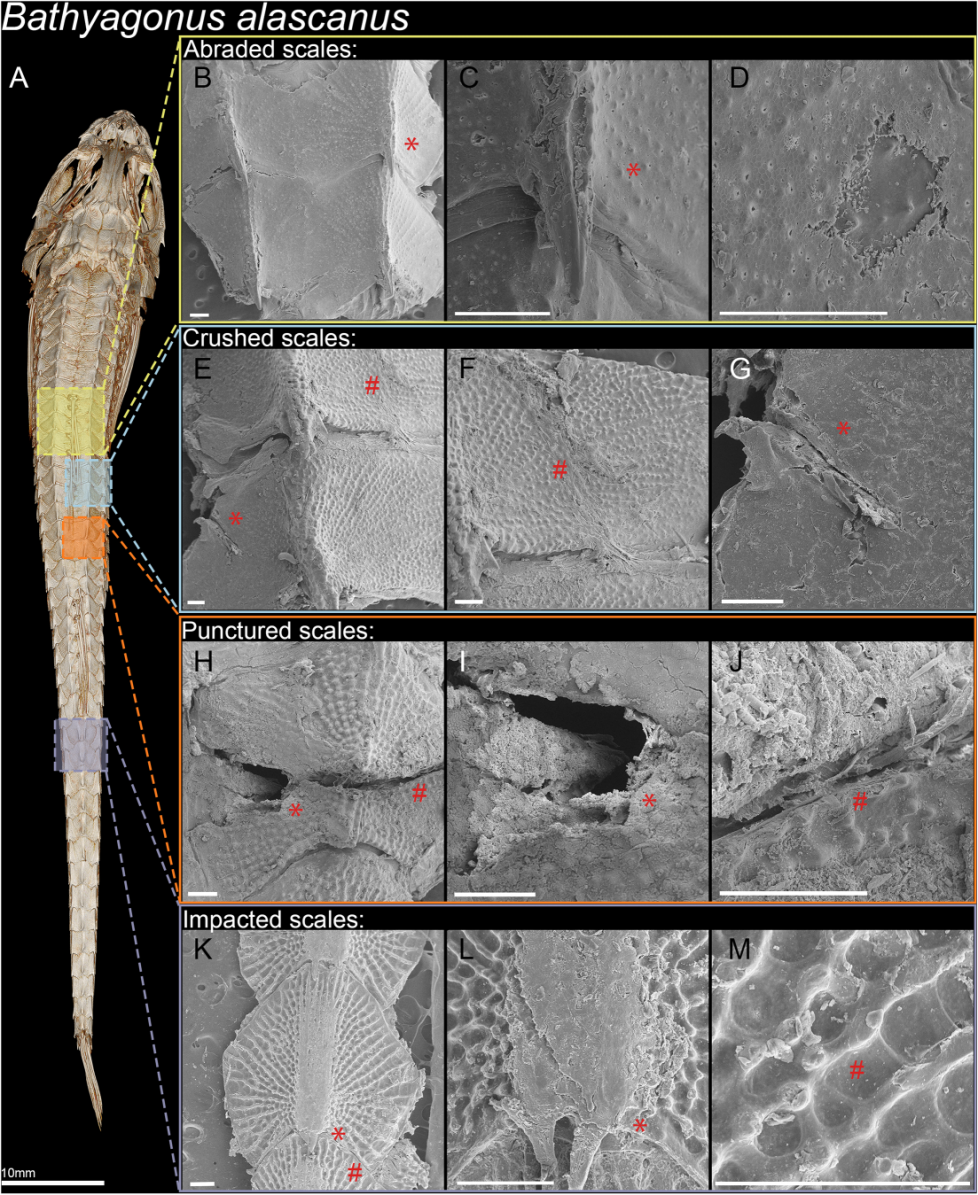
Scanning electron microscopy images from dissected dorsal and lateral *B. alascanus* scales following damage testing. SEM image scale bars measure 250 μm, asterisks and hashtags denote damages repeated between pictures. (A) Dorsal view μCT scan of a *B. alascanus*. Highlighted are the scales dissected for SEM imaging. Row one: (B) Dorsal scales smoothened following sand abrasion, losing outer dermal layers, (D) external ridges, and (C) spine height due to erosion. Row two: (E) Dorsal scales cracked from crushing tests, (F) one showing a large curved crack, (G) the other scale a smaller longitudinal crack. Row three: (H) A dorsal scale punctured between the dorsal fins, showing (I) a large cavern from the site of puncture and (J) a fracture traveling across the plate surface. Row four: Dorsal scales chipped and eroded from rock impact tests. (K) Spines are chipped and (L) flattened, (M) while ridges are mostly maintained but show some chip damage.

μCT scans of the rock-tumbled specimens showed the highest points on plate exteriors had been chipped and fragmented. This effect was not uniform—some spines were nearly flattened, while others were intact. A dorsal sample of three plates was dissected from the DPT region for SEM, which confirmed that the spines were flattened (Fig. 7K), showing chips in the rest of the plate (Fig. 7L). Ridges on the plates were unevenly damaged (Fig. 7M), some of the higher points in the ridges showed similar impact damage as the spines.

μCT scan visualizations from sand-eroded specimens showed that the bony exterior appeared to be intact, with no acute damage. However, SEM images provided finer detail of the plate surfaces, revealing plate exteriors had been smoothed (Fig. B, C, and D). The ridges and bumps normally present were polished away, leaving a smooth outer surface. The outermost dermal layers were usually eroded evenly, but in some specimens, abrasion spared parts of the inner layers. Only anterior plates, taken from dorsal rows between the dorsal fins, were imaged using SEM. Rock-tumbled specimens were less uniformly damaged, with higher points bearing the brunt of damage as compared to the evenly distributed abrasion that the sanded plates experienced. However, both treatments showed damage in just 30 min of tumbling.

## Discussion

### Poacher armor exhibits regional specialization

Different armor morphologies will offer better protection against different force modes. The armor of *B. alascanus* has specialized functionality across the body, possibly driven by morphological differences in plate size, shape, and degree of overlap. The ratio of armor to soft tissue volume also changes from anterior to posterior due to the fusiform shape of the body. In the anterior region, the plates form an octagonal cross section with a low armor-to-volume ratio, while, toward the posterior region, the cross section shifts to a hexagonal shape with a much higher armor-to-volume ratio. It is possible that crushed specimens experience failures from buckling, which will occur easier when there are more plate rows, as the joints where adjacent plates meet will fail rapidly compared to a solid plate. Under compressive forces from puncture or crushing, plates interlock more tightly due to increased imbrication in the hoop direction (around the body circumference). Once fully locked, the armor, together with the vertebrae and ribs, appears to act as a strut system that dissipates force across multiple plates, indicating an adaptation for resisting buckling rather than localized bending. Structural interlocking is particularly effective in the tail, where the hexagonal geometry provides high compressive strength and enhanced energy dissipation, features commonly used in both natural and engineered systems (Hu and Yu 2010; Verbrugghe et al. 2023). The reduced number of sides in a hexagonal cross section also allows the tail to reach a fully interlocked state more quickly, reducing opportunities for plates to slip or shift (Currey 2002). In poachers, this results in lower variability in the failure stress when the crushing force is applied to the flat sides (rather than the vertices) of the octagon or hexagon (Fig. 3). Furthermore, the doubled dorsal and ventral plate rows at anterior locations fail rapidly due to the vertex formed where they meet, while the fusion of these plates at the tail increases resistance to crushing forces. Once fully interlocked, crushing forces cause failure at the weakest point, regardless of where force is applied (Fig. 5) (Currey 2002). These points will additionally fail faster in the octagonal geometry where there are more vertices that increase the effective length of each side and decrease the buckling strength of the cross-section.

While a hexagonal cross-section is better for resisting a crushing compressive force, the octagonal shape offers a better defense against puncture. Puncture forces are more concentrated which causes localized failures or fractures where force is applied. In an octagon, more rows means more work must be exerted to fully compress the system before critical failure will occur. Higher degrees of plate imbrication distribute the force over a wider area as the plates interact (Zhu et al. 2013; Martini and Barthelat 2016). In poachers, puncture forces acting on the anterior end often contact two overlapping scales, rather than a single plate. These regions of overlapping plates resist puncture significantly better than individual plates (Martini and Barthelat 2016; Lowe et al. 2021). Although increased overlap reduces flexibility and adds weight, it provides enhanced protection for vital organs located at the front of the body.

### Plates reflect functional tradeoffs and damage history

Beyond the arrangement of the armor as a whole, the morphology of individual plates also plays a critical role in preventing system wide damage. Each plate is roughly hexagonal in shape and features dense radiating ridges that extend outward from a central spine. In specimens subjected to crushing, fractures consistently occurred along this central spine, suggesting failure due to bending of individual plates. These fractures were typically parallel to the central spine, in turn, preserving the integrity of the rest of the plate (Figs. 4D and 5A). Under a localized puncture force, cracks radiated from the point of puncture until they were contained by adjacent mineralized ridges, preventing damage from propagating across the plate. This suggests that crushing forces are channeled along existing structural plate axes, while puncture forces may propagate unpredictably, possibly indicating anisotropy in both material and structural layout. Such patterns may reflect structural adaptations to contain cracks such as in the striped bass (*Morone saxatilis*) where punctured scales showed controlled fractures that aligned with the underlying collagen fibril orientation, greatly contributing to puncture resistance (Zhu et al. 2012). The dense central spines could also serve a protective role similar to the internal scute (osteogenically developed scales, as opposed to odontogenic) ridges found in the three-stripe corydoras (*Corydoras trilineatus*), which shield the center of the scute, an area not covered by overlapping scutes, from puncture (Sire et al. 2009; Lowe et al. 2021). In this way, the shape and material distribution of individual poacher armor plates resist damage from compressive forces, either diffuse (crushing) or localized (puncture).

Plate morphology may also reflect developmental or evolutionary responses to repeated damage. For instance, adult poachers have spineless ventral plates, which could arise from gradual wear or from new plates forming without spines in regions where abrasion and impact damage is consistently high (Kruppert et al. 2020). The absence of spines likely reflects a tradeoff between protection and durability. Investment into ventral spine mineralization would be rapidly lost from collision against rocks in the strong subtidal currents. After 30 min of tumbling in a small space with little room for rocks to accelerate, many dorsal and lateral spines were visibly chipped or flattened, resembling the naturally smooth ventral plates. In high-energy subtidal environments, where ventral surfaces routinely contact rocky substrates, this effect would be amplified. A similar pattern has been observed in Pacific spiny lumpsucker armor, where impact (collision) damage spalls armor, while abrasion wears away high protrusions on the armor surface (Woodruff et al. 2022). If such damage occurs rapidly *in-situ*, it raises the possibility that armor plates—particularly in high-impact zones—undergo continual remodeling or partial regeneration. While poacher armor is heavily mineralized, some bony plates in fishes are known to remodel at the surface through osteocytic activity or dermal resorption (e.g., scales in ganoid fishes or denticles in elasmobranchs) (Bereiter-Hahn and Zylberberg 1993). The mineralized armor plates likely reflect an investment in resistance to impact damage that is largely dependent on hardness and Young’s modulus (Amini and Miserez 2013; Kruppert et al. 2020; Zheng et al. 2023). The radiating ridges of each plate may act as sacrificial barriers, absorbing damage and protecting the underlying surfaces. The flatter thick ridges provide a much sturdier barrier to constant abrasion than thin protruding spines that are easily lost.

### Armor function is shaped by ecology

While the rows and plates of poacher armor show impressive regional specialization and mechanical adaptability, they may be better suited for minimizing damage than fully preventing it during predation. Our specimens withstood only slightly more than half the estimated crushing force of a red rock crab (*Cancer productus*) and, though the anterior region is better suited to resist puncture, many piscivorous fish attack prey with puncturing teeth from the tail end (Taylor 2000; Muruga et al. 2022). These observations suggest poacher armor may primarily function to delay or reduce damage during an attack, buying time for escape. Perhaps their tightly imbricated armor stiffens the body, increasing locomotor efficiency or allowing energy storage for increased acceleration (Tytell et al. 2010).

Although we understand how different regions of the poacher body respond to different mechanical stresses, we have not done a detailed morphological analysis of the variation across these regions. There are shifts in plate shape, thickness, and spine density across the body, suggesting region-specific adaptations. Biological materials often employ gradients in material properties such as mineralization that can dissipate impact stress and stop crack propagation (He et al. 1994; Amini and Miserez 2013). Mineralization within and across individual poacher plates is not yet known but may reflect the damage types they encounter daily. Our species of interest, *B. alascanus*, invests relatively little in armor relative to other poachers, so perhaps selection for protection has been relaxed (Kruppert et al. 2020). Alternatively, this lower investment may reflect an optimization toward flexibility and energy efficiency—traits that are difficult to reconcile with extensive mineralized armor.

## Data Availability

The data underlying this article are available in Github and CT data will be available on MorphoSource.
